# Modulating Tumor-Associated Macrophage Polarization by Synthetic and Natural PPARγ Ligands as a Potential Target in Breast Cancer

**DOI:** 10.3390/cells9010174

**Published:** 2020-01-10

**Authors:** Giulia Gionfriddo, Pierluigi Plastina, Giuseppina Augimeri, Stefania Catalano, Cinzia Giordano, Ines Barone, Catia Morelli, Francesca Giordano, Luca Gelsomino, Diego Sisci, Renger Witkamp, Sebastiano Andò, Klaske van Norren, Daniela Bonofiglio

**Affiliations:** 1 Department of Pharmacy, Health and Nutritional Sciences, University of Calabria, 87036 Arcavacata di Rende (CS), Italy; giu.gionfriddo@gmail.com (G.G.); pierluigi.plastina@unical.it (P.P.); giusy.augimeri@gmail.com (G.A.); stefania.catalano@unical.it (S.C.); cinzia.giordano@unical.it (C.G.); ines.barone@unical.it (I.B.); catia.morelli@unical.it (C.M.); francesca.giordano@unical.it (F.G.); luca.gelsomino@unical.it (L.G.); diego.sisci@unical.it (D.S.); sebastiano.ando@unical.it (S.A.); 2 Division of Human Nutrition and Health, Wageningen University, 6700 AA Wageningen, The Netherlands; renger.witkamp@wur.nl

**Keywords:** tumor associated macrophages, breast tumor microenvironment, peroxisome proliferator-activated receptor gamma, *n*-3 polyunsaturated fatty acids, docosahexaenoyl ethanolamine (DHEA), docosahexaenoyl serotonin (DHA-5-HT), rosiglitazone

## Abstract

Activation of peroxisome proliferator-activated receptor gamma (PPARγ) elicits anti-proliferative effects on different tumor cells, including those derived from breast cancer. PPARγ is also expressed in several cells of the breast tumor microenvironment, among which tumor associated macrophages (TAMs) play a pivotal role in tumor progression and metastasis. We explored the ability of synthetic and natural PPARγ ligands to modulate TAM polarization. The ligands included rosiglitazone (BRL-49653), and two docosahexaenoic acid (DHA) conjugates, *N*-docosahexaenoyl ethanolamine (DHEA) and *N*-docosahexaenoyl serotonin (DHA-5-HT). Human THP-1 monocytic cells were differentiated into M0, M1 and M2 macrophages that were characterized by qRT-PCR, ELISA and western blotting. A TAM-like phenotypic state was generated by adding two different breast cancer cell conditioned media (BCC-CM) to the cultures. Macrophages exposed to BCC-CM concomitantly exhibited M1 and M2 phenotypes. Interestingly, rosiglitazone, DHEA and DHA-5-HT attenuated cytokine secretion by TAMs, and this effect was reversed by the PPARγ antagonist GW9662. Given the key role played by PPARγ in the crosstalk between cancer cells and TAMs in tumor progression, its activation via endogenous or synthetic ligands may lead to novel strategies that target both epithelial neoplastic cells and the tumor microenvironment.

## 1. Introduction

Despite major advances in prevention, screening and treatment, breast cancer remains the leading cause of cancer incidence and mortality in women worldwide, with 2.1 million new cases, accounting for almost one in four diagnosed cancer cases in 2018 [1]. Risk factors include genotypic predisposition, hormonal and environmental factors, reproductive history, age and lifestyle [2,3,4]. Regarding lifestyle, several lines of evidence underline the importance of dietary factors as an important contributor to risk and disease progression for several types of cancer, including breast carcinoma [5]. Specifically, a Western diet which is among others characterized by high intakes of *n*-6 polyunsaturated fatty acids (PUFAs), and lower intake of *n*-3 PUFAs has been suggested to play a role in carcinogenesis and cancer outcomes [6,7]. In line with this, an increasing number of studies point towards the importance of two main dietary *n*-3 PUFAs, eicosapentaenoic acid (EPA) and docosahexaenoic acid (DHA), in breast cancer prevention and treatment [8,9,10,11,12]. These molecules serve as structural components of cellular membranes and are assumed to exert antineoplastic activities through alteration of membrane fluidity and cell surface receptor function, modulation of cyclooxygenase (COX) activity and an attenuation of increased cellular oxidative stress [13]. In addition, EPA and DHA show direct affinity for peroxisome proliferator-activated receptor gamma (PPARγ) [14], which is well-known for its metabolic functions [15], but also for its involvement in inflammation [16,17]; and in tumor suppressor action by the virtue of promoting growth inhibition, apoptosis, cell cycle arrest and re-differentiation in several malignancies [18,19,20,21]. Besides exerting direct effects, DHA and EPA are endogenously converted to a plethora of bioactive molecules in a condition and tissue-specific manner [22,23,24]. This includes the formation of conjugates with biological amines leading to endocannabinoid-like structures. An example is the conversion of dietary DHA into its ethanolamide, *N*-docosahexaenoyl ethanolamine (DHEA), which has been identified in many tissues and in plasma, and whose release was shown to be induced during inflammatory conditions [25,26,27,28]. Our research group also showed the antiproliferative effects of DHEA on breast cancer cell lines through PPARγ activation [29]. Moreover, it has been demonstrated that DHEA displays marked anti-inflammatory properties by inhibiting the formation of eicosanoids via COX-2 in macrophages [30,31]. DHA can also be metabolized into other endocannabinoid congeners of which the potential physiological activities often continue to be discovered. One of these bioactive fatty acid mediators is the serotonin conjugate of DHA (*N*-docosahexaenoyl serotonin, DHA-5-HT). Previously, DHA-5-HT has been identified in intestinal tissues, and its levels were markedly influenced by intake of *n*-3 PUFAs [32]. More recently, DHA-5-HT has been found also to modulate inflammation in macrophages and blood mononuclear cells with profound effects on several key mediators [33,34]. However, little is known about its full biological significance or pharmacological potential.

Macrophages are an intrinsically heterogeneous population which display high plasticity. When challenged, tissue-resident and circulating monocyte-derived macrophages alter their basal states by a process known as activation or “polarization” and acquire diverse supportive functions specialized to different tissue compartments. In the context of cancer, tumor-associated macrophages (TAMs) are particularly abundant within tumors and promote cancer cell proliferation, immunosuppression and angiogenesis in support of tumor growth and metastasis [35]. Macrophages display divergent phenotypes, classically designated as pro-inflammatory [lipopolysaccharide (LPS)-induced macrophages, M1] and anti-inflammatory (IL-4-stimulated macrophages, M2). In situations of immunological homeostasis, these sub-populations were assumed to be in balance. However, more recent studies showed that regarding tumor development and progression, this picture is more complex. As it has been demonstrated, M1 macrophages have the capacity to kill and remove tumor cells, in line with their primary physiological function in phagocytosis [36]. At the same time, M2 cells drive tumor development in both primary and metastatic sites through their contributions to basement membrane breakdown and deposition, angiogenesis, recruitment of leukocytes and overall immune suppression [37,38]. Remarkably, macrophages within the tumor microenvironment are not limited to M1 or M2 states, but instead may reside in-between or off this spectrum. Removal of all macrophage populations regardless of polarization state has emerged as a potential therapeutic option, as there is a significant reduction in both primary and metastatic tumorigenesis [39]. 

Based on the anti-inflammatory and antineoplastic properties found for conjugates of the *n*-3 long-chain PUFAs [28,29,30,31,33,34,40,41,42], we investigated the effects of two DHA metabolites, DHEA and DHA-5-HT, and the synthetic PPARγ ligand rosiglitazone (BRL-49653), on modulating TAM polarization induced by breast cancer cell conditioned media (BCC-CM). Our results demonstrate that culturing M0 macrophages in medium to which BCC-CM has been added induces a differentiation into a TAM-like phenotypes, which is attenuated by natural and synthetic PPARγ agonists, highlighting the potential of such compounds from a pharmacological or nutritional perspective as TAM-targeting agents.

## 2. Materials and Methods

### 2.1. Reagents

Phorbol 12-myristate 13-acetate (PMA), lipopolysaccharide (O111:B4; LPS), rosiglitazone (BRL-49653) and GW9662 (GW) were purchased from Sigma-Aldrich (Schnelldorf, Germany). *N*-docosahexaenoyl serotonin (DHA-5-HT) and *N*-docosahexaenoyl ethanolamine (DHEA) were from Cayman Chemical (Ann Arbor, MI, USA). Interleukin-4 (IL4) was purchased from R&D Systems (Abingdon, UK).

### 2.2. Cell Culture

Human THP-1 monocytic cell line, human ERα-positive MCF7 and the triple-negative (ER-, PR- and HER2-negative) MDA-MB-231 breast cancer epithelial cells were acquired from American Type Culture Collection where they were authenticated, stored according to supplier’s instructions and used within 4 months after recovery of the frozen aliquots. THP-1 cells were cultured in Roswell Park Memorial Institute (RPMI)-1640 medium (Lonza, Verviers, Belgium), supplemented with 10% fetal calf serum (FCS, Lonza) and 1% penicillin-streptomycin (Life Technologies, Carlsbad, CA, USA) at 37 °C in a humidified 5% CO_2_ atmosphere. MCF7 cells were cultured in DMEM (Life Technologies) supplemented with 10% fetal bovine serum (FBS, Life Technologies), 1 mg/mL penicillin-streptomycin and 0.01 mg/mL insulin (Sigma Aldrich) at 37 °C in a humidified 5% CO_2_ atmosphere. MDA-MB-231 cells were cultured in DMEM/F-12 plus Glutamax (Life Technologies) containing 10% FBS and 1 mg/mL penicillin-streptomycin at 37 °C in a humidified 5% CO_2_ atmosphere. 

### 2.3. Macrophage Differentiation and Polarization 

To obtain differentiated M0 macrophages, one million THP-1 cells were seeded in 6-well plates in RPMI media plus 100 nM PMA for 24 h followed by 1 day of rest in medium without PMA. Characteristic morphologic changes of monocyte-to-macrophage differentiation were observed and photographed by an Olympus IX-70 microscope. Macrophages were stimulated for 6 h with 10 ng/mL LPS or with 20 ng/mL IL4 for 72 h to generate M1 or M2 macrophages, respectively.

### 2.4. Macrophage and Breast Cancer Cell Culture System

MCF7 and MDA-MB-231 breast cancer cells (BCC) were cultured until 80–90% confluence and then incubated with serum-free media for 48 h. The BCC-conditioned media (CM) were collected, centrifuged at 2000× *g* at 4 °C for 10 min to remove cell debris and stored at −80 °C. Mixed-medium culture systems were established by incubation of differentiated M0 macrophages with BCC-CM in a 1:1 ratio with RPMI medium for 72 h. To evaluate the effects of PPARγ stimulation, BCC-CM was added to macrophages in the presence or absence of rosiglitazone, DHEA, DHA-5HT and GW9662 as described. 

### 2.5. RNA Purification and Quantitative Reverse-Transcription Real-Time PCR

Total RNA was extracted using TRIzol (Invitrogen, Breda, The Netherlands), as suggested by the manufacturer. RNA (1 μg/sample) was reverse transcribed to give complementary DNA (cDNA) using the reverse-transcription system from Promega (Leiden, The Netherlands). cDNA was amplified by qRT-PCR using the master-mix Sensimix SYBR (Bioline Reagents Ltd., London, UK) on a CFX Real Time System apparatus (Bio-Rad, Veenendaal, The Netherlands). Samples were analyzed in duplicate and mRNA expression levels of the different genes were normalized to RPS27A2 or RNA18S and calculated as described [43]. Primers are listed in Table S1 (Supplementary Material).

### 2.6. Cytokine Array

Human XL Cytokine Array Kits, obtained from R&D Systems (Minneapolis, MN, USA), were used to analyze the secreted proteins in the conditioned medium derived from M1 and M2 macrophages, according to the manufacturer’s recommendations. The intensity of selected spots was quantified using Image Studio Lite Version 5.2 (Licor, Lincoln, NE, USA).

### 2.7. Enzyme-Linked Immunosorbent Assay (ELISA) 

Levels of Interleukin-6 (IL6) and Interleukin-1 receptor antagonist (IL1Ra) were measured in supernatants from macrophages using human ELISA kits according to manufacturer’s instructions (R&D Systems). Specifically, in mixed-medium culture systems, cells were maintained in serum-free medium for another 24 h, and supernatants were collected and used for analyses.

### 2.8. Western-Blotting Analysis

Macrophages were washed twice with PBS and lysed in RIPA buffer (50 mM Tris-HCl, 150 mM NaCl, 1% Nonidet P-40, 0.5% sodium deoxycholate, 2 mM sodium fluoride, 2 mM EDTA and 0.1% SDS). Total protein extracts (30 μg) were resolved on 10% SDS-polyacrylamide gel, as described [44]. After blocking, proteins were probed with anti-PPARγ (sc7196), anti-GAPDH (sc25778) and anti-β−Actin (sc69879) (Santa Cruz Biotechnology, Santa Cruz, CA, USA), antibodies, and with anti-STAT3 (9139s) (Cell Signaling Technology, Danvers, MA, USA) antibody, overnight, and were detected by using a chemiluminescence (ECL) system (Bio-Rad USA). For a set of experiments, images were acquired using Odissey FC (Licor).

### 2.9. Flow Cytometry

THP-1 cells were seeded in 60 mm dishes, differentiated and treated as indicated. Cells were washed with cold PBS; detached with versine; pelleted; resuspended in a total of 100 µL of cold PBS containing 5 µL of PE anti-CD80 antibody (number 557227) (Becton Dickinson Italia, MI, Italy) or FITC anti-CD206 antibody (number 321103) (BioLegend, San Diego, CA, USA); and incubated 15 min at room temperature in the dark. After incubation, cells were washed with 1 × PBS and centrifuged at 500 × *g* for 5 min and then re-suspended in 500 μL of 1 × PBS. Cells were analyzed by FACScan flow cytometer (Becton Dickinson, Mountain View, CA, USA) and the data acquired using CellQuest software (version 3.3). Unstained cells were used to determine the background autofluorescence to set the negative population allowing cells stained with anti-CD80 (or anti-CD206) antibody to be visualized.

### 2.10. Phagocytosis Assay

THP-1 cells were seeded in 2-well chamber slides, differentiated and treated as indicated. Macrophages were then assessed for phagocytic activity using the Phagocytosis Assay Kit (Cayman Chemical, Ann Arbor, MI, USA) as recommended by the manufacturer. Briefly, cells were incubated for two hours with the latex beads-rabbit IgG-FITC complex (1:250) followed by cell fixation with 4% paraformaldehyde. Cells were washed with assay buffer and then counterstained with 4’,6-diamidino-2-phenylindole (DAPI). Fluorescence was photographed with OLYMPUS BX51 microscope, 20X objective. Pixel density of FITC labeled beads above threshold standardized between coverslips was normalized to number of nuclei, using the DAPI staining method, obtained using ImageJ software (version 1.52, NIH, Bethesda, MD, USA). 

### 2.11. Cytotoxicity Assays

Potential cytotoxicity effects of MCF7 and MDA-MB-231 breast cancer cells (BCC)-conditioned media (CM), rosiglitazone, DHA-5HT and DHEA were evaluated by measuring lactate dehydrogenase (LDH) leakage using a Cytotoxicity Detection Kit (Roche Applied Science, Almere, The Netherlands), as previously reported [40]. Briefly, after incubating macrophages with BCC-CM alone or in combination with the compounds for 72 h, 100 µL supernatants were mixed with enzyme reagents (diaphorase/NAD mixture, 250 µL) and dye solutions (iodotetrazolium chloride and sodium lactate, 11.25 mL). After incubating for 30 min at 25 °C, the absorbance was measured at 492 nm. Cytotoxicity values were expressed as percentages with respect to cells treated with Triton X-100 (set as 100%) at the end of the experiments.

### 2.12. Statistical Analysis

Data were presented as means ± SDs. Experimental data were analyzed for statistical significance by one-way ANOVA test and Student’s *t*-test using the GraphPad Prism7 software program. Differences with *p* < 0.05 (*), *p* < 0.005 (**), *p* < 0.0005 (***), *p* < 0.0001 (****) were considered statistically significant. 

## 3. Results

### 3.1. Optimized Technical Conditions for Human THP-1 Macrophage Polarization

**Figure 1:**
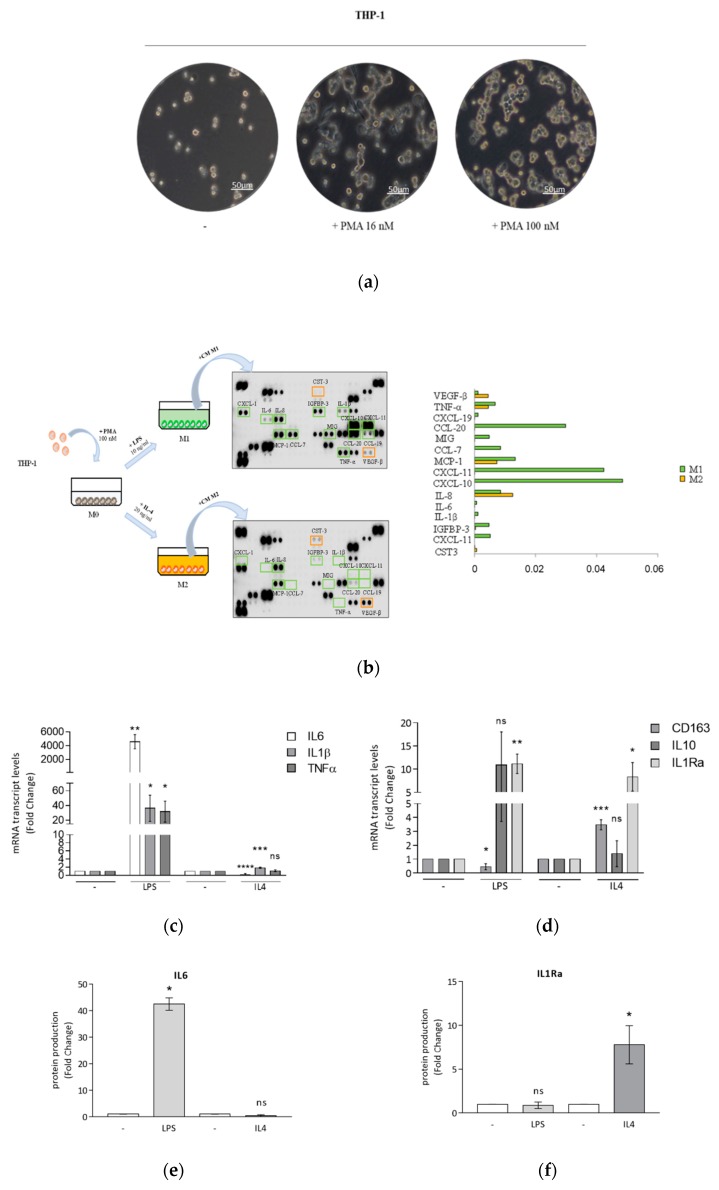

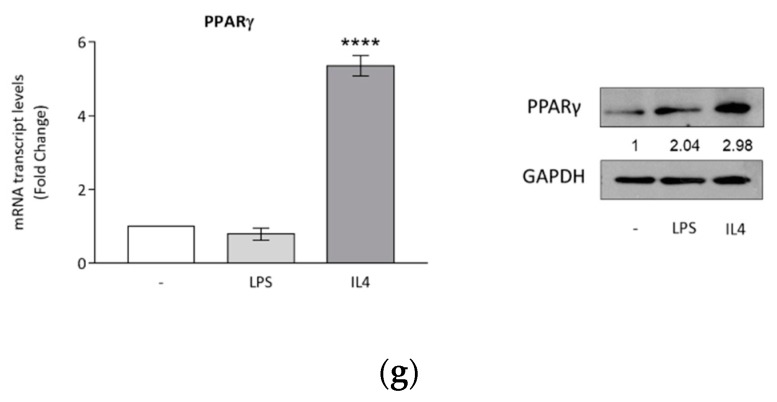
Differentiation and polarization of THP-1 monocytes. (**a**) Representative bright field images by optical microscopy of morphological changes in THP-1 monocytes (-) differentiated into mature macrophages M0 in the presence of 16 nM or 100 nM PMA for 24 h. The scale bar represents 50 μm. (**b**) Macrophages derived from the stimulation with PMA 100 nM (M0), after replacement of PMA-containing medium with fresh medium (without PMA) for 1 day, were treated with LPS 10 ng/mL or IL4 20 ng/mL for 24 h to obtain M1 and M2 macrophages, respectively. Human cytokine arrays were used for the detection of secreted proteins in conditioned media derived from M1 and M2 macrophages after 24 h of polarization. Raw numerical densitometry data were extracted and the background subtracted. Results were shown as mean pixel density. Real-time RT-PCR of M1 markers IL6, IL1β, TNFα (**c**) and M2 markers CD163, IL10 and IL1Ra (**d**) in M0 macrophages (-) treated with LPS 10 ng/mL for 6 h or IL4 20 ng/mL for 72 h. Each sample was normalized on its RPS27A mRNA content. ELISA analyses of IL6 (**e**) and IL1Ra (**f**) proteins were performed in conditioned media of polarized macrophages. Values represent means ± SDs of three different experiments, each performed with duplicate samples. Results are expressed as fold changes compared to M0 macrophages (-). (**g**) Real-time RT-PCR and immunoblotting of PPARγ in M0 macrophages (-) treated with LPS 10 ng/mL or IL4 20 ng/mL for 24 h. Each sample was normalized on its 18S RNA content. Glyceraldehyde-3-phosphate dehydrogenase (GAPDH) was used as a loading control. Numbers below the blots represent the average fold change between PPARγ and GAPDH protein expression versus M0 macrophages. Results are expressed as fold changes compared to M0 macrophages, * *p* < 0.05, ** *p* < 0.005, *** *p* < 0.0005, **** *p* < 0.0001, ns = not significant.

The human monocytic THP-1 cell line, derived from a patient with acute leukemia, provides a valuable model for understanding macrophage biology [45]. Despite the wide use of this cell line, macrophage differentiation and/or polarization protocols differ highly among studies. Thus, we first assessed which strategies were the most effective in producing populations of pro-inflammatory M1- and anti-inflammatory M2-polarized THP-1 cells. Based on the literature [45], monocytes were stimulated with low (16 nM) or high (100 nM) phorbol 12-myristate 13-acetate (PMA) concentrations to induce monocyte differentiation into mature macrophages (M0). After 24 h of culture with both PMA concentrations, followed by replacement of PMA-containing medium with fresh medium (without PMA) for 1 day, cells adhered to the dish bottom and had the typical hallmarks of M0 macrophages, represented by cell adhesion and spread morphology (Figure 1a). Then, the differentiation protocol was optimized for the levels of lipopolysaccharide (LPS) and interleukin-4 (IL4) needed to obtain distinct M1 and M2 phenotypes. In THP-1 cells treated with 100 nM PMA and then stimulated by 10 ng/mL LPS or 20 ng/mL IL4 for 24 h, we detected a clear distinctive protein secretion pattern of cytokines in conditioned media (Figure 1b). In addition, we found that the maximal phenotypic changes were obtained upon 6 and 72 h of exposure to LPS and IL4, respectively. Using the above selected experimental conditions, mRNA expression levels of M1 typical markers were significantly increased upon LPS treatment (Figure 1c), while mRNA expression levels of M2 markers were strongly upregulated in cells stimulated by both LPS and IL4 (Figure 1d). Next, we analyzed specific protein levels of M1 and M2 macrophage markers secreted in the conditioned media. These data revealed an increased production of interleukin-6 (IL6) in LPS-treated cells, whereas interleukin-1 receptor antagonist (IL1Ra) levels were significantly elevated only in IL4-stimulated macrophages (Figure 1e).

Given the documented role of peroxisome proliferator-activated receptor gamma (PPARγ) in the regulation of macrophage maturation [46], we evaluated mRNA and protein levels of PPARγ, by using qRT-PCR and immunoblotting analyses, respectively, in macrophages differentiated and polarized according to our protocol. As expected, PPARγ was expressed at protein levels in both classically activated M1 and alternatively activated M2 phenotypes (Figure 1g), while at the mRNA level it was up-regulated only in IL4-stimulated cells.

### 3.2. Breast Cancer Cells Drive Macrophage Polarization of THP-1 Cells

**Figure 2:**
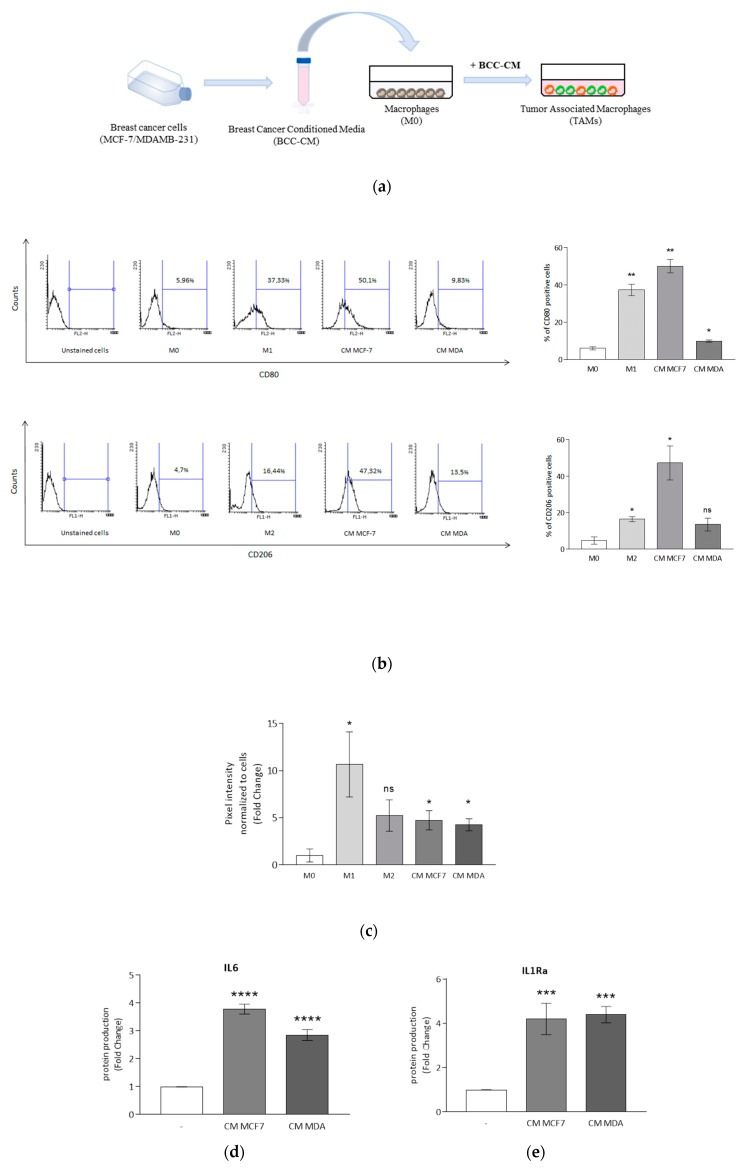
Macrophage polarization induced by breast cancer cells. (**a**) Conditioned media from breast cancer cells (BCC-CM) incubated in serum-free medium for 48 h were collected and added to M0 macrophages for 72 h to obtain tumor-associated macrophages (TAMs). (**b**) Flow cytometry analyses of M1 marker CD80 and M2 marker CD206 in M0, M1 and M2 macrophages and in M0 cells incubated with CM MCF7 or CM MDA for 72 h. The bars represent the percentages of positive cells. * *p* < 0.05, ** *p* < 0.005 versus M0 macrophages. (**c**) Phagocytic activity of M0, M1 and M2 macrophages and M0 cells incubated with CM MCF7 or CM MDA for 72 h following incubation with latex beads conjugated with FITC-IgG for 2 h. Pixel intensity of FITC labeled beads was normalized to number of cells and results are expressed as fold change respect to M0. ELISA analyses of IL6 (**d**) and IL1Ra (**e**) in M0 macrophages (-) incubated with CM MCF7 or CM MDA for 72 h. Values represent means ± SDs of three different experiments, each performed with duplicate samples. The results are expressed as fold change with respect to differentiated cells. * *p* < 0.05, ** *p* < 0.005, *** *p* < 0.0005, **** *p* < 0.0001, ns = not significant.

Macrophage polarization occurs in response to local micro-environmental molecules and signals in a wide spectrum of physiological and pathological processes, including cancerous diseases [47]. In breast carcinoma, tumor-associated macrophages (TAMs), regardless of polarization state, retain the capacity for plasticity, including the ability to switch between phenotypes as a function of micro-environmental cues [48]. To mimic the breast tumor microenvironment, M0 macrophages were cultured for 72 h with breast cancer conditioned media (BCC-CM) obtained from two different types of breast cancer cells, MCF7 (CM MCF7) and MDA-MB-231 (CM MDA) cells (Figure 2a). To evaluate the polarization state, flow cytometry analyses and phagocytosis assays were performed. We found that CM MCF7 was able to induce a significant upregulation of both CD80 and CD206 (M1 and M2 markers, respectively), whereas in the case of CM MDA the upregulation was observed only for CD80 (Figure 2b). In addition, both BCC-CM types increased phagocytotic capacity compared with M0 macrophages (Figure 2c). Moreover, gene expression and protein secretion data from co-cultured cells were analyzed in the same experimental conditions. While the response for M1 and M2 mRNA expression markers displayed a high data dispersion, which was reflected in high standard errors when averaging independent experiments (Figure S1a,b in Supplementary Material), we observed a significant BCC-induced upregulation of both IL6 and IL1Ra proteins, that mirrored phenotypic changes associated with M1 and M2 macrophage polarization (Figure 2d,e). 

### 3.3. Rosiglitazone Negatively Affects Cytokine Secretion Induced by Breast Cancer Cell Conditioned Media 

**Figure 3:**
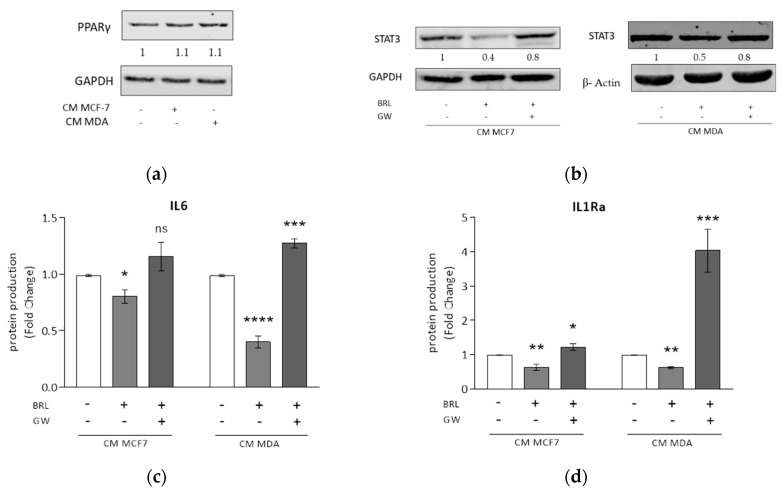
Rosiglitazone antagonizes macrophage cytokine secretion induced by conditioned media derived from MCF7 and MDA-MB-231 breast cancer cells. (**a**) Immunoblotting of PPARγ in M0 macrophages (-) incubated with CM MCF7 or CM MDA for 72 h. Glyceraldehyde-3-phosphate dehydrogenase (GAPDH) was used as loading control. The blot is representative of three independent experiments, while the numbers below the blots represent the average fold change between PPARγ and GAPDH protein expression with respect to M0 macrophages. (**b**) Immunoblotting of STAT3 in M0 macrophages incubated with CM MCF7 or CM MDA and treated with rosiglitazone (BRL) 10 μM alone or in combination with GW9662 (GW) 10 μM for 72 h. GAPDH or β-actin was used as the loading control. Each blot is representative of three independent experiments, while the numbers below the blots represent the average fold change between STAT3 and GAPDH or β-actin protein expression with respect to vehicle-treated cells. ELISA analyses of IL6 (**c**) and IL1Ra (**d**) proteins in M0 macrophages incubated with CM MCF7 or CM MDA, and treated with BRL 10 μM alone or in combination with GW 10 μM for 72 h. Data are expressed as means ± SDs of three independent experiments, each performed with duplicate samples. The results are expressed as fold changes with respect to vehicle-treated cells (-). * *p* < 0.05, ** *p* < 0.005, *** *p* < 0.0005, **** *p* < 0.0001, ns = not significant.

In the breast tumor microenvironment, several cells, including TAMs, express PPARγ [47]. In line with these findings, we observed in TAMs, generated by stimulation with CM from MCF7 and MDA-MB-231 (MDA) breast cancer cells, the expression of PPARγ (Figure 3a). Subsequently, we explored the effects of PPARγ activation on macrophage polarization induced by breast cancer cells. First, to test the potential cytotoxicity of PPARγ ligands, LDH release in cell culture media was measured as an indicator for cell leakage. We used the synthetic and specific PPARγ agonist rosiglitazone at a concentration of 10 μM for 72 h in M0 macrophages cultured with either CM MCF7 or CM MDA, since cell cytotoxicity values were similar compared to the respective CM control (Table S1 in Supplementary Material). Then, we found that PPARγ activation with rosiglitazone inhibited the signal transducer and activator of transcription (STAT) 3 protein, a crucial crossroad for cytokine production, in M0 macrophages cultured with either CM MCF7 or CM MDA. This effect was reversed by the specific PPARγ antagonist GW9662 (Figure 3b). Based on those observations, we explored the effects of the inhibition of this signaling pathway on interleukin secretion. As expected, treatment with rosiglitazone was able to decrease IL6 and IL1Ra production by macrophages exposed to BCC-CM of both tumor cell lines. The effects induced by rosiglitazone were completely abrogated upon co-treatment with GW9662 (Figure 3c,d). However, the percentages of positive cells for both M1 and M2 cell surface markers of macrophages treated with BCC-CM in combination with PPARγ agonist rosiglitazone did not substantially change, as revealed by flow cytometry analysis (Figure S2 in Supplementary Material). Similarly, gene expression for a set of validated immunophenotypic markers for the two polarization states using TAMs treated with rosiglitazone displayed a high variability when assessing statistical significance, which did not mirror the same effects obtained by cytokine secretions (Figure S3 in Supplementary Material).

### 3.4. DHA Conjugates DHEA and DHA-5-HT Counteract TAM Cytokine Secretion 

**Figure 4:**
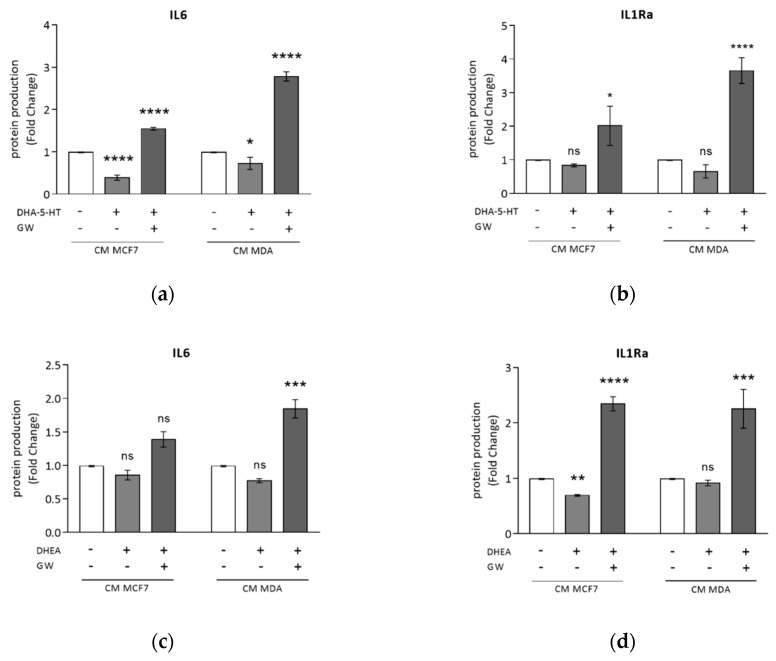
DHA-5-HT and DHEA counteract TAM cytokine secretion induced by MCF7 and MDA-MB-231 breast cancer cell conditioned media. ELISA analyses of IL6 (**a**,**c**) and IL1Ra (**b**,**d**) in M0 macrophages incubated with CM MCF7 or CM MDA, and treated with DHA-5-HT 1 μM (a,b), DHEA 5 μM (**c** and **d**) or a combination with GW9662 (GW) (a–d) for 72 h. Data are expressed as means ± SDs. Each experiment was performed three times with duplicate samples. The results are expressed as fold changes with respect to vehicle-treated cells (-).* *p* < 0.05, ** *p* < 0.005, *** *p* < 0.0005, **** *p* < 0.0001, ns = not significant.

In earlier studies we found that DHA-derived compounds inhibit breast cancer progression and development through PPARγ activation [29,41]. Thus, in this study we investigated the effects of DHA conjugates with ethanolamine and serotonin, DHEA and DHA-5-HT, respectively, in modulating macrophage polarization induced by BCC-CM. Since DHEA at 5 μM and DHA-5-HT at 1 μM concentrations were not toxic to TAMs, as revealed by LDH release (Table S1 in Supplementary Material), we evaluated protein amounts secreted by TAMs generated by exposure to CM MCF7 or CM MDA in presence of these compounds for 72 h. As shown in Figure 4a, DHA-5-HT significantly reduced IL6 production by both TAMs, which was completely upregulated by the PPARγ antagonist GW9662. A similar but not significant decrease with DHA-5-HT was observed with IL1Ra levels, whereas GW9662 treatment resulted in a marked and significant upregulation (Figure 4b). DHEA stimulation strongly downregulated IL1Ra secretion only in macrophages co-cultured with CM MCF7; once again, GW9662 was able to reverse these effects resulting in a higher production of cytokines (Figure 4c,d). Flow cytometry analysis did not show any change in the percentage of positive cells for cell surface markers in macrophages treated with BCC-CM in combination with both compounds (Figure S2 in Supplementary Material). Regarding gene expression patterns related to macrophage polarization, mRNA levels of M1 markers were reduced following DHEA and DHA-5-HT treatments, while none of the M2 markers were changed (Figure S4 in Supplementary Material). 

## 4. Discussion

Smoldering inflammation has been considered a hallmark of cancer with an important role in tumor initiation and progression [49]. Clinically, the presence of macrophages/TAMs within primary tumors has been shown to correlate with poorer prognosis in almost all tumors. Higher numbers of M1 macrophages within these sites are associated with better prognosis, whereas a balance towards more M2 macrophages predicts poor outcomes [50,51]. However, studies on macrophage–cancer cell interactions have emphasized that macrophages are heterogeneous, plastic cells with different/opposite functions and cytokine production in response to various micro-environmental switching signals [47,52]. In this study, we first documented that human differentiated THP-1 macrophages exposed to media from MCF7 or MDA-MB-231 breast cancer cells displayed a TAM-like phenotype with features of both M1 and M2 polarized cells, as confirmed by flow cytometry assay. In addition, functional studies revealed an increased phagocytic activity after induction with both BCC-CM. 

The THP-1 cell line has been described to be a suitable model to study macrophage differentiation, functions and responses to external stimuli from the micro and macro-environment [45,53,54].

In line with previous findings [55], our data suggest that soluble interactions sustain the breast cancer-macrophage communication. In particular, conditioned media from human breast adenocarcinoma cells were found to contain soluble factors playing a crucial role for the recruitment and adhesion of myeloid cells to the tumor, including macrophage inflammatory protein-1β (MIP-1β), vascular endothelial growth factor-α (VEGF-α), IL-8, IFN-γ and granulocyte colony-stimulating factor (G-CSF) [56]. Similarly, it has been reported for co-cultures with adipocytes [57], endothelial cells [58] and mesenchymal stem cells [59] that cytokine levels increase when breast cancer cells interact with stroma. In turn, TAMs acting as the cellular source of inflammatory mediators perpetuate the local inflammatory milieu, which through paracrine and autocrine signaling networks further promotes breast cancer development [48]. Thus, strategies aiming to remove macrophages and/or alter macrophage phenotypes promise therapeutic benefits. 

In our search for novel TAM-modulating agents, we tested the effects of natural and synthetic PPARγ agonists on the capacity of the breast cancer cell secretome to modulate macrophage polarization. We observed that the synthetic specific PPARγ agonist rosiglitazone reduced secretion of M1 pro-inflammatory and pro-tumor M2-cytokines. Moreover, the two conjugates of *n*-3 PUFA with ethanolamine and serotonin, DHEA and DHA-5-HT, respectively, exerted similar inhibitory effects without affecting macrophage polarization. In particular, our data indicate that the regulation of cytokine production seems to occur on the protein level rather than on mRNA level.

Dietary *n*-3 PUFAs have well-established anti-tumorigenic effects in in vitro and in vivo models of breast cancer [6,12]. Moreover, *n*-3 PUFAs have been reported to reduce cancer-cachexia [60,61,62,63]. In breast cancer cells, DHA strongly reduces cell viability and DNA synthesis and promotes cell death via apoptosis [9]. The proposed mechanisms include the ability of DHA to decrease the total amount of and to alter the size of lipid rafts in breast cancer cells, disrupting membrane signaling involved in the regulation of cell survival and proliferation and sensitizing cells to apoptosis [64]. The protective effects of *n*-3 PUFA result in reduced breast cancer incidence, growth, multiplicity and metastasis in rodent models of breast cancer [64]. Furthermore, in a mouse model of obese postmenopausal breast cancer, *n*-3 PUFA supplementation reduced mammary adipose tissue inflammation and markers of inflammatory M1 macrophage infiltration [65] which were associated with reduced tumor burden, indicating that the inflammatory microenvironment promotes tumorigenesis and that *n*-3 PUFA directly antagonizes this process. Similarly, it has been reported that *n*-3 PUFA supplementation led to up-regulation of the expression of several genes involved in cell cycle regulation in overweight humans, highlighting the anti-tumorigenic effects of such compounds [66]. 

Importantly, many biological effects of DHA are known to be exerted by molecules derived from them which are endogenously synthesized in a tissue-specific manner and at least partly triggered by inflammatory signals [25,26,27]. We have previously demonstrated, for the first time, that DHEA through PPARγ activation induces cell growth inhibition, triggering autophagy in breast cancer cells [28]. Moreover, DHEA has been reported to possess anti-inflammatory- and (or) general immune-modulating properties [25,26,27,28], and to exert antitumor effects in prostate cancer cells [67]. Also DHA-5-HT has been shown to modulate inflammation in macrophages by reducing levels of key mediators involved in cytokine signaling pathways. These pathways are receiving much attention because of their involvement in several chronic disorders, including cancer, making DHEA and DHA-5-HT potentially attractive pharmacological tools.

Interestingly, our data revealed that the anti-inflammatory action of DHEA and DHA-5-HT in TAMs, as shown by a lower production of IL-6 cytokine, was prevented by the PPARγ antagonist GW9662, suggesting the potential involvement of PPARγ. Increasing evidence points towards an important role of PPARγ in cancer. Although it has been reported that activation of PPARγ maintained cancer stem cell properties in ErbB2-positive breast cancer cells [68], a large body of evidence has shown that PPARγ hinders tumor development and progression, in most cases through modulation of differentiation, proliferation, apoptosis and motility of cancer cells via a variety of molecular pathways [18,19,20,21]. In addition to regulating the oncogenic activities of cancer cells, PPARγ can control the tumor microenvironment, affecting multiple aspects of the cancer-associated inflammatory responses. Particularly, agonist activation of PPARγ, whose expression increases upon macrophage and monocyte activation [69], is known to suppress the production of inflammatory mediators such as IL-6 [16]. The negative regulation of inflammatory responses is mediated by the inhibition of transcription factors, such as members of the signal transducer and activator of transcription (STAT) protein family, often occurring via an interaction with PPARγ that sequesters them from their response elements, preventing inflammatory responses [70]. We also observed a reduction of STAT3 expression induced by ligand activated PPARγ along with a decreased production of IL-6 in TAMs. This highlights a link between PPARγ, inflammation and cancer. Furthermore, PPARγ also participates in controlling alternative activation of monocytes and macrophages [71]. A positive correlation between the expression of M2 markers and PPARγ was supported by the ability of Th2 cytokines such as IL4 to enhance PPARγ expression in monocytes/macrophages and of the IL-4-STAT6-PPAR-γ signaling axis in monocytes to control their differentiations into alternatively activated macrophages [71,72].

In our attempts to optimize experimental conditions to obtain monocyte-to-macrophage differentiation and polarization, we observed an increased mRNA and protein PPARγ expression in M2 compared to that in M0 macrophages, while no differences were observed in our generated TAMs. More interestingly, synthetic and natural PPARγ agonists significantly reduced the production of the cytokine IL1Ra, simultaneously attenuating both M1 and M2 macrophage phenotypes, which are known to promote a pro-tumorigenic milieu in the breast tumor microenvironment. 

In parallel to modulating M2 polarization of TAMs, several strategies have been developed to interrupt metabolic pathways for energy and metabolite production able to support their specialized cellular activities, including the modulation of PPARγ-Gpr132-lactate signaling [48]. PPARγ agonists have been successfully used to desensitize TAMs to lactate stimulation with antitumor effects in breast cancer [73].

## 5. Conclusions

The role of the tumor microenvironment is increasingly acknowledged for cancers of the breast and other tissues. TAMs are one of the most abundant stromal components, playing a key role in tumor progression. Thus, strategies aimed to specifically target TAMs may represent an important approach to improving therapeutic outcome. Endogenous or synthetic PPARγ agonists may offer leads to novel strategies that target both epithelial neoplastic cells and the tumor microenvironment. As a next step, studies using monocyte-derived macrophages and autologous cells from breast cancer patients are warranted to further explore the therapeutic potential of our findings.

## Supplementary Materials

The following are available online at https://www.mdpi.com/2073-4409/9/1/174/s1. Figure S1: Real-time RT-PCR of M1 and M2 markers in TAMs. Figure S2. Flow cytometry analyses of cell surface M1 and M2 markers in TAMs with rosiglitazone, DHA-5-HT or DHEA. Figure S3: Real-time RT-PCR of M1 and M2 markers in TAMs treated with rosiglitazone. Figure S4: Real-time RT-PCR of M1 and M2 markers in TAMs treated with DHEA or DHA-5-HT. Table S1: Oligonucleotide primers used in this study. Table S2: Lactate dehydrogenase (LDH) release into supernatant media of BCC-CM, alone and with rosiglitazone, DHEA and DHA-5-HT.
