# Supplementary material for: Modulating Tumor-Associated Macrophage Polarization by Synthetic and Natural PPARγ Ligands as a Potential Target in Breast Cancer
Source: Cells. 2020 Jan 10;9(1):174. doi: 10.3390/cells9010174 (PMC7017381; doi:10.3390/cells9010174)
Supplement: Supplementary file 1 [file cells-09-00174-s001.pdf]

Modulating Tumor-Associated Macrophage polarization by synthetic and natural PPARγ ligands as a potential target in breast cancer

Giulia Gionfriddo [1,#], Pierluigi Plastina [1,#], Giuseppina Augimeri [1], Stefania Catalano [1], Cinzia Giordano [1], Ines Barone [1], Catia Morelli [1], Francesca Giordano [1], Luca Gelsomino [1],Diego Sisci [1], Renger Witkamp [2], Sebastiano Andò [1], Klaske van Norren [2,*], Daniela Bonofiglio [1,*]

[1] Department of Pharmacy, Health and Nutritional Sciences, University of Calabria, 87036 Arcavacata di Rende (CS), Italy; giu.gionfriddo@gmail.com (G.G.); pierluigi.plastina@unical.it (P.P); giusy.augimeri@gmail.com (G.A.); stefania.catalano@unical.it (S.C.); cinzia.giordano@unical.it (C.G.); ines.barone@unical.it (I.B.); catia.morelli@unical.it (C.M.); francesca.giordano@unical.it (F.G.); luca.gelsomino@unical.it (L.G.); diego.sisci@unical.it (D.S.); sebastiano.ando@unical.it (S.A.); daniela.bonofiglio@unical.it (D.B.)

[2] Division of Human Nutrition and Health, Wageningen University, 6700 AA Wageningen, The Netherlands; renger.witkamp@wur.nl (R.W.); klaske.vannorren@wur.nl (K.v.N.)

# G.G. and P.P. equally contributed to this work.

* Correspondence: daniela.bonofiglio@unical.it; Tel.: +39-0984-496208 (D.B.); klaske.vannorren@wur.nl @wur.nl; Tel.: +31-0317-485093 (K.v.N.)

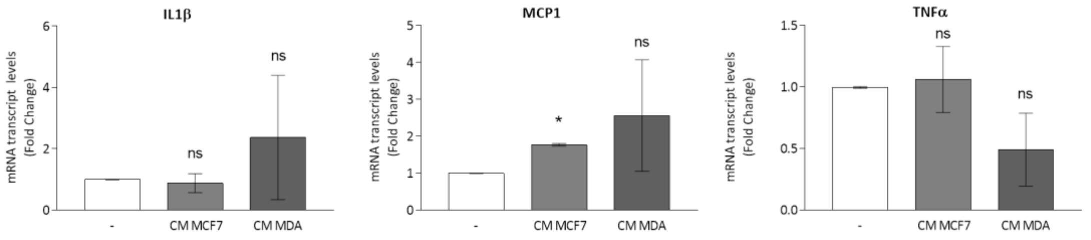

(**a**)

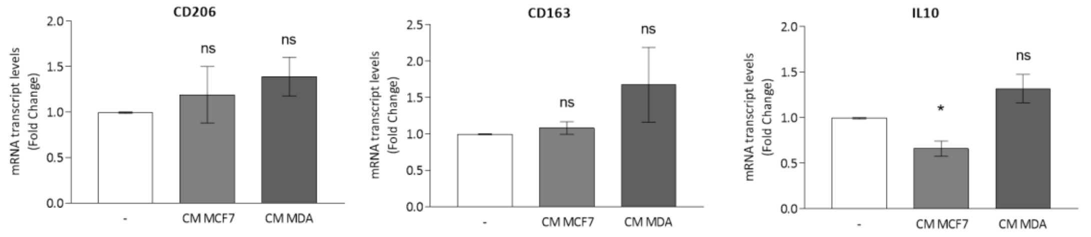

(**b**)

Figure S1. Real-time RT-PCR of M1 and M2 markers in TAMs. Real-time RT-PCR of M1 markers IL1β, MCP1, TNFα (a) and M2 markers CD206, CD163, IL10 (b) in M0 macrophages (-) incubated with CM MCF7 or CM MDA. Each sample was normalized on its RPS27A mRNA. Values represent means ± SD of three different experiments, each performed with duplicate samples. The results are expressed as fold change respect to differentiated cells. *P < 0.05. ns= not significant.

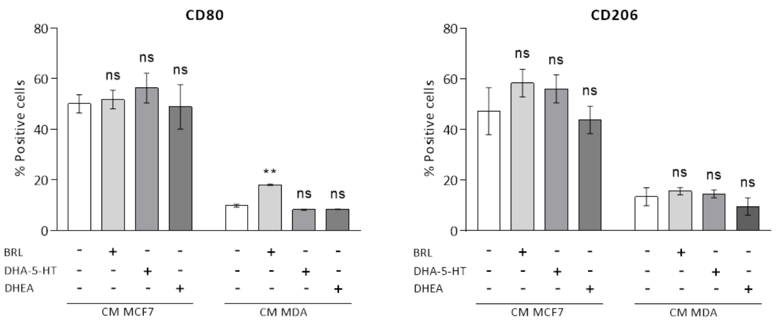

(**a**) (**b**)

Figure S2. Flow cytometry analyses of cell surface M1 and M2 markers in TAMs with rosiglitazone, DHA-5-HT or DHEA. Flow cytometry analyses of M1 marker CD80 (a) and M2 marker CD206 (b) in M0 macrophages incubated with CM MCF7 or CM MDA and rosiglitazone (BRL) 10 μM, DHA-5-HT 1 μM or DHEA 5 μM for 72 h. Data are expressed as means ± SD. Each experiment was performed two times with duplicate samples. The results are expressed as percentage of positive cells respect to vehicle-treated cells (-).** $P < 0.005$, ns= not significant.

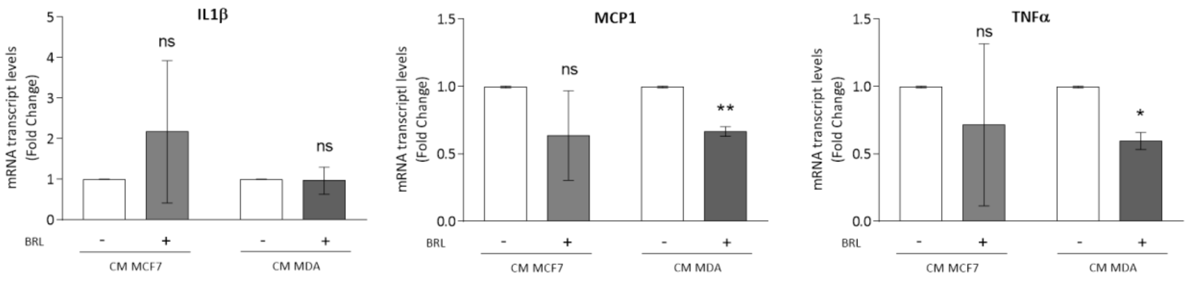

(**a**)

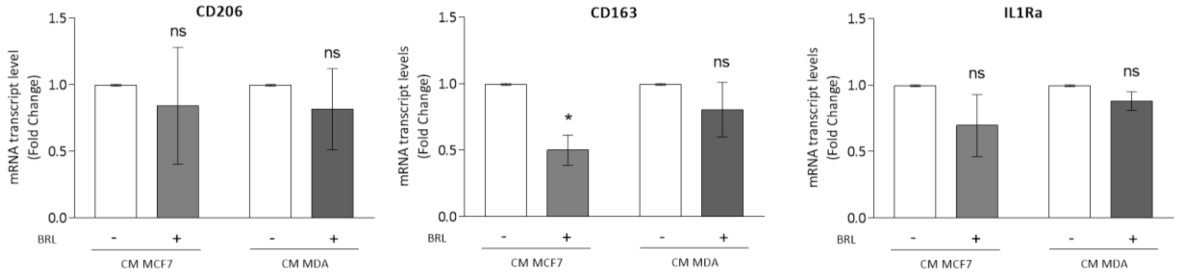

(**b**)

Figure S3. Real-time RT-PCR of M1 and M2 markers in TAMs treated with rosiglitazone. Real-time RT-PCR of M1 markers IL1β, MCP1, TNFα (a) and M2 markers CD206, CD163, IL1Ra (b) in M0 macrophages incubated with CM MCF7 or CM MDA and treated with rosiglitazone (BRL) 10 μM for 72 h. Each sample was normalized on its RPS27A mRNA. Data are expressed as means ± SD. Each experiment was performed two times with duplicate samples. The results are expressed as fold change respect to vehicle-treated cells (-). *$P < 0.05$, ** $P < 0.005$, ns= not significant.

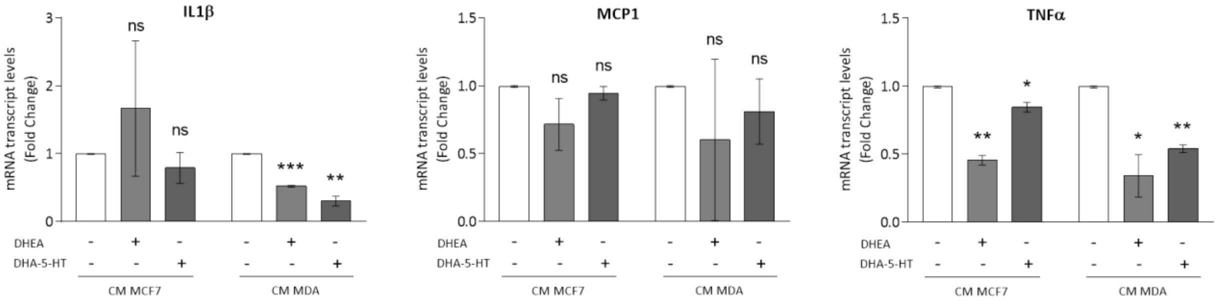

(**a**)

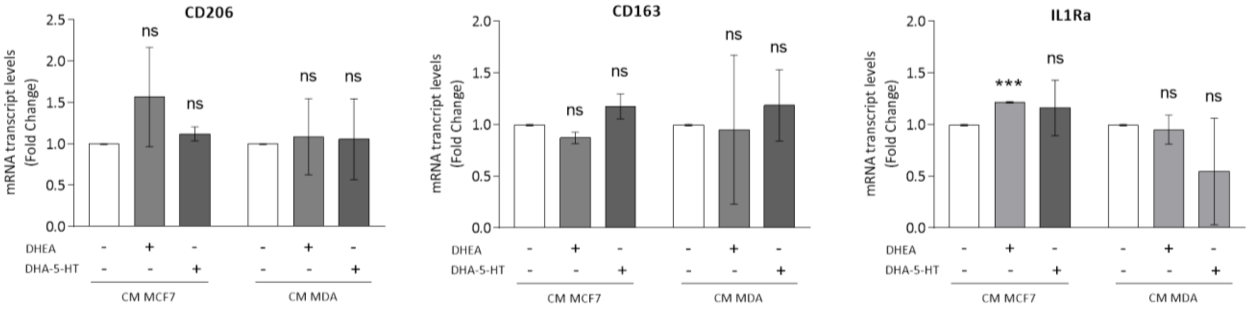

(**b**)

Figure S4. Real-time RT-PCR of M1 and M2 markers in TAMs treated with DHEA or DHA-5-HT. Real-time RT-PCR of M1 markers IL1β, MCP1, TNF*α* (a) and M2 markers CD206, CD163, IL1Ra (b) in M0 macrophages incubated with CM MCF7 or CM MDA and DHEA 5 μM or DHA-5-HT 1 μM for 72 h. Each sample was normalized on its RPS27A mRNA. Data are expressed as means ± SD. Each experiment was performed two times with duplicate samples. The results are expressed as fold change respect to vehicle-treated cells (-). *$P < 0.05$, ** $P < 0.005$, ***$P < 0.0005$, ns= not significant.

Table S1. Oligonucleotide primers used in this study.

Gene name	Gene symbol		Primer Sequences
Interleukin 6	*IL6*	Forward	5′- AACCTGAACCTTCCAAAGATGG -3′
		Reverse	5′- TCTGGCTTGTTCCTCACTACT-3′
Interleukin 1 beta	*IL1β*	Forward	5′- CACGATGCACCTGTACGATCA-3′
		Reverse	5′- GTTGCTCCATATCCTGTCCCT-3′
Monocyte Chemoattractant Protein 1	*MCP1*	Forward	5′- CCCCAGTCACCTGCTGTTAT-3′
		Reverse	5′- AGATCTCCTTGGCCACAATG-3′
Tumor Necrosis Factor alpha	*TNFα*	Forward	5′- ATGAGCACTGAAAGCATGATCC-3′
		Reverse	5′- GAGGGCTGATTAGAGAGAGGTC-3′
Mannose Receptor C-type 1, MRC1	*CD206*	Forward	5′- GGGTTGCTATCACTCTCTATGC-3′
		Reverse	5′- TTTCTTGTCTGTTGCCGTAGTT-3′
CD163 molecule	*CD163*	Forward	5′- ACTTGAAGACTCTGGATCTGCT-3′
		Reverse	5′- CTGGTGACAAAACAGGCACTG-3′
Interleukin 1 Receptor Antagonist	*IL1RA*	Forward	5′- GCCTCCGCAGTCACCTAAT-3′
		Reverse	5′- TCCCAGATTCTGAAGGCTTG-3′
Interleukin 10	*IL10*	Forward	5′- ACTTTAAGGGTTACCTGGGTTGC-3′
		Reverse	5′- TCACATGCGCCTTGATGTCTG -3′
Peroxisome Proliferator Activated Receptor gamma	*PPARγ*	Forward	5′- GGCTTCATGACAAGGGAGTTTC-3′
		Reverse	5′- AACTCAAACTTGGGCTCCATAAAG-3′
Ribosomal Protein S27A	*RPS27A*	Forward	5′- GTTAAGCTGGCTGTCCTGAAA-3′
		Reverse	5′- CATCAGAAGGGCACTCTCG-3′
18S Ribosomal RNAs	*RNA18S*	Forward	5′-CGGCGACGACCCATTCGAAC-3′
		Reverse	5′-GAATCGGAACCCTGATTCCCCGTC-3′

Table S2. Lactate dehydrogenase (LDH) release into supernatant media of BCC-CM, alone and with rosiglitazone, DHEA and DHA-5-HT. Lactate dehydrogenase (LDH) release into supernatant media after 72 hour treatment with MCF-7 and MDA-MB-231 breast cancer cell conditioned media (CM), alone and with rosiglitazone (BRL), DHEA and DHA-5-HT. Absorbance of reduced formazan dye at 490 nM was normalized to the dispersion-media control. Triton X-100 was used as a positive control and represents 100% of LDH release.

**Treatment**		**Concentration (μM)**	**Cytotoxicity (%)**
Triton-X-100		-	100
MCF-7-CM		-	24±4
MDA-MB-231-CM		-	32±6
MCF-7-CM	BRL	10	32±9
	DHEA	5	27±2
	DHA-5-HT	1	27±5
MDA-MB-231-CM	BRL	10	37±10
	DHEA	5	27±3
	DHA-5-HT	1	33±12
